# TB sequel: incidence, pathogenesis and risk factors of long-term medical and social sequelae of pulmonary TB – a study protocol

**DOI:** 10.1186/s12890-018-0777-3

**Published:** 2019-01-07

**Authors:** Andrea Rachow, Olena Ivanova, Robert Wallis, Salome Charalambous, Ilesh Jani, Nilesh Bhatt, Beate Kampmann, Jayne Sutherland, Nyanda E. Ntinginya, Denise Evans, Knut Lönnroth, Stefan Niemann, Ulrich E. Schaible, Christof Geldmacher, Ian Sanne, Michael Hoelscher, Gavin Churchyard

**Affiliations:** 1 Division of Infectious Diseases and Tropical Medicine, Medical Centre of the University of Munich (LMU), Munich, Germany; 2 German Center for Infection Research (DZIF), Partner Site Munich, Munich, Germany; 3 The Aurum Institute, Johannesburg, South Africa; 4 Instituto Nacional de Saúde (INS), Ministry of Health, Maputo, Mozambique; 5 Medical Research Council Unit The Gambia, Banjul, The Gambia; 6 Department of Medicine, Imperial College London, London, UK; 7 NIMR - Mbeya Medical Research Centre, Mbeya, Tanzania; 8 Health Economics and Epidemiology Research Office, Department of Internal Medicine, School of Clinical Medicine, Faculty of Health Sciences, University of the Witwatersrand, Johannesburg, South Africa; 9 Department of Public Health Sciences, Karolinska Institutet, Stockholm, Sweden; 10 Research Center Borstel, Borstel, Germany; 11 Clinical HIV Research Unit, Department of Internal Medicine, School of Clinical Medicine, Faculty of Health Sciences, University of the Witwatersrand, Johannesburg, South Africa; 12 School of Public Health, University of Witwatersrand, Johannesburg, South Africa; 13 Advancing Care and Treatment for TB/HIV, South African Medical Research Council, Parktown, Johannesburg, South Africa

**Keywords:** Tuberculosis, Tuberculosis outcome, Sequelae, Lung function, Lung impairment, Patient costs, Study protocol, Cohort, Africa, Risk factors, Treatment outcome

## Abstract

**Background:**

Up to fifty percent of microbiologically cured tuberculosis (TB) patients may be left with permanent, moderate or severe pulmonary function impairment. Very few studies have systematically examined pulmonary outcomes in patients to understand the pathophysiologic basis and long-term socio-economic consequences of this injury. The planned multi-country, multi-centre observational TB cohort study, aims to advance the understanding of the clinical, microbiological, immunological and socio-economic risk factors affecting long-term outcome of pulmonary TB. It will also determine the occurrence of reversible and irreversible socio-economic consequences to patients, their households and the health sector related to pulmonary TB disease and its treatment.

**Methods:**

We will enrol up to 1.600 patients with drug sensitive and multidrug-resistant pulmonary TB who are treated according to the local standard of care by the respective National TB Program. Recruitment is taking place at the time of TB diagnosis at four African study clinics located in The Gambia, Mozambique, South Africa and Tanzania. The primary outcome is the proportion of TB patients with severe lung impairment measured by spirometry at 24 months after TB treatment initiation. Biological samples, including sputum, urine and blood, for studying host- and pathogenic risk factors will be collected longitudinally and examined in a nested case-control fashion. A standardized quality of life questionnaire will be used together with a novel version of WHO’s generic patient cost instrument which has been adapted for the longitudinal study design.

**Discussion:**

This study is an integral part of an overall strategy to fill a knowledge gap needed to improve TB treatment outcomes globally. The main scientific goal is to identify the major pathogenic mechanisms associated with poor TB treatment outcomes, so that such pathways can be interrupted to avert long term TB sequelae. National as well as supra-national stakeholders and decision makers have been integrated early in the study planning process to inform future treatment guidelines and national health policies.

**Trial registration:**

ClinicalTrials.gov: NCT03251196, August 16, 2017.

**Electronic supplementary material:**

The online version of this article (10.1186/s12890-018-0777-3) contains supplementary material, which is available to authorized users.

## Background

Tuberculosis (TB) remains one of the world’s deadliest communicable diseases. In 2016, an estimated 10.4 million people developed TB and 1.3 million died from the disease [1]. TB related morbidity and mortality remain particularly high in African countries, mainly due to the impact of HIV, sustained poverty and food insecurity as well as due to treatment challenges including the rise in drug-resistant TB [2].

For the past 40 years, treatment success in tuberculosis has been defined as the eradication of active infection whilst preventing resistance and recurrence, achieved through multidrug antimicrobial treatment. Current estimates of the global TB disease burden include incidence and prevalence of active TB, TB death rates, and disability-adjusted years of life (DALY) lost due to active TB, but do not consider DALYs lost due to long-term disability due to TB sequelae or reduced longevity in patients considered cured [3, 4]. However, accumulating evidence indicates that permanent lung injury due to TB is frequent and substantial. In one study in Papua New Guinea, the mean FEV1 (the maximal volume of air exhaled in the first second after a full inspiration), was reduced to 64% of that of healthy controls at the time of TB diagnosis [5]. FEV1 improved by 11% after 2 months of TB treatment, but did not further improve subsequently. At the conclusion of successful anti-microbial treatment, 27% of TB patients still had moderate or severe impairment of pulmonary function. [5]. Another study from India found severe and irreversible obstructive pulmonary ventilation defects in one-third of TB patients one year after completion of TB treatment [6]. A literature review of South African studies showed that lung function impairment and chest symptoms were consistently associated with pulmonary TB [7]. In the United States, studies demonstrated that even persons cured from TB have considerably shortened life expectancy [5, 6]. There is limited evidence on pulmonary function impairment in African populations outside South Africa and presently no estimates of the impact of TB on life expectancy in African patients. Further, associated risk factors contributing to permanent lung function impairment after TB are unknown.

### Clinical, environmental and behavioural risk factors

The risk of developing TB is up to 30 times greater in people living with HIV than among those without HIV infection [8]. TB patients with advanced AIDS are at increased risk of immune reconstitution inflammatory syndrome (IRIS) and death. The long-term effects of HIV and antiretroviral treatment on lung function, however, are not known. Diabetes mellitus is also recognized as a TB risk factor and the African region is experiencing an increasing prevalence of diabetes alongside other non-communicable diseases [9–11]. However, little is known about the potential impact of diabetes on long-term TB outcomes. Other risk factors that can potentially have long term effects include pre-existing pulmonary conditions like Chronic Obstructive Pulmonary Disease (COPD) and asthma, and associated risk factors such as smoking, indoor air pollution and harmful alcohol use [12, 13]. Malnutrition is also an important risk factor for TB [14], however, the association with sequelae after treatment has not been studied.

### Host-immune response and associated risk factors

It is well known that host responses to Mycobacterium tuberculosis (MTB) also contribute to lung pathology through excessive induction of the inflammatory pathway. However, whether the systemic host response before and after TB treatment initiation can be used to predict pathology and treatment outcomes is largely unknown. Blood and sputum-derived neutrophil activation and inflammatory mediators will be examined in order to elucidate the predominant pathway of lung inflammation in TB and inform on future host-adjunctive therapy strategies. For example, polymorpho-nuclear neutrophilic granulocytes have been associated with active tuberculosis and the failure to control the infection, and likely contribute to exacerbated pathology and long term sequelae and lung function loss [15–17]. Additionally, there are very few studies that have assessed specific host biomarkers in clinical research studies, such as the T-cell activation marker-tuberculosis (TAM TB) assay [18], which have shown a correlation with diagnosis and treatment response in TB [19].

### Pathogen diversity and microbial risk factors

There is increasing evidence that genomic diversity of the MTB complex influences important patho-biological properties, such as transmissibility and pathogenicity, and affects host-immune responses and clinical manifestations [20, 21]. Moreover, microbial factors are recognized as important determinants of microbiologic outcomes such as treatment failure or relapse. Baseline drug resistance can affect the risk of new (acquired) resistance and subsequent treatment failure. For some drugs, such as rifampin, this effect can be profound [22]. Sputum bacillary burden at baseline and culture status after 2 months of treatment affect relapse risk [23]. However, very little is known about the contributions of all these and other microbial factors, such as strain genetic background or the presence of mixed infections, on long-term pulmonary function outcomes.

### Socio-economic determinants and costs of tuberculosis

Several previous studies have documented high patient costs before patients are diagnosed and during TB treatment [24, 25], where patients may lose about half of their household income due to TB. Loss of income and direct expenses may result in poorer treatment outcomes, including increased risk of TB infection and progression, the development of drug resistance and long term pulmonary impairment. The WHO End TB Strategy has included an microeconomic target, that no TB patient or affected household should experience catastrophic expenditure or costs related to TB illness and TB care [26], and a generic protocol for the measurement of catastrophic costs due to TB has been developed [27]. Measuring catastrophic cost is a useful indicator of equality in advancing towards universal health coverage. Although patient costs during diagnosis and TB treatment has been documented there is little evidence on the costs and socioeconomic consequences of TB after treatment has been completed and how they are associated to different clinical TB outcomes. In addition, the published literature contains surprisingly little evidence about the costs to the health systems of treating TB and TB sequelae, especially in Africa.

### Study aim

TB Sequel project aims to: i) advance understanding of the clinical, microbiological, immunological and socio-economic risk factors affecting or predicting the long-term pulmonary function outcome; ii) determine costs on patient and health system level related to TB disease, TB treatment and TB sequelae.

## Methods/design

### Study partners and setting

The TB Sequel cohort study is conducted by the TB Sequel consortium including a number of research institutions based in Africa and Europe (Additional file 1). The Division of Infectious Diseases and Tropical Medicine at the University of Munich (LMU), Germany and The AURUM Institute in Johannesburg, South Africa coordinate the project. The TB Sequel cohort study will recruit TB patients in four African countries of which three belong to high TB burden countries. In Johannesburg, South Africa, the study is conducted by the University of Witwatersrand (WITS) at the Clinical HIV Research Unit (CHRU) located at the Helen Joseph Hospital that serves an urban area with approximately one million inhabitants. In Mbeya, Tanzania, the study takes place at the National Institute of Medical Research - Mbeya Medical Research Centre (NIMR-MMRC). The NIMR-MMRC is located within the same premises of the Mbeya Zonal Referral Hospital (MZRH), one of the four largest referral hospitals in Tanzania, which serves a total population of about 500.000 inhabitants. In Maputo, Mozambique, the Instituto Nacional de Saúde (INS) is conducting the TB Sequel cohort study at two research units, first located in Mavalane Health Centre (City of Maputo) serving approximately 620.000 inhabitants, and second, located in Machava General Hospital (Province of Maputo) where the majority of MDR-TB patients of Maputo are treated. In The Gambia, this study is conducted by the Medical Research Council (MRC) Unit The Gambia in the Greater Banjul area, where two thirds of all TB patients diagnosed in the country present. The Research Center Borstel (FZB) in Germany is responsible for MTB isolates typing and contributes to host responses studies including training of African researchers. The socio-economic sub-study is being led by the Health Economics and Epidemiology Research Office (HE^2^RO) at WITS University in South Africa in collaboration with the Department of Public Health Sciences at Karolinska Institutet, Sweden and WHO Geneva.

### Study design

**Fig. 1:**
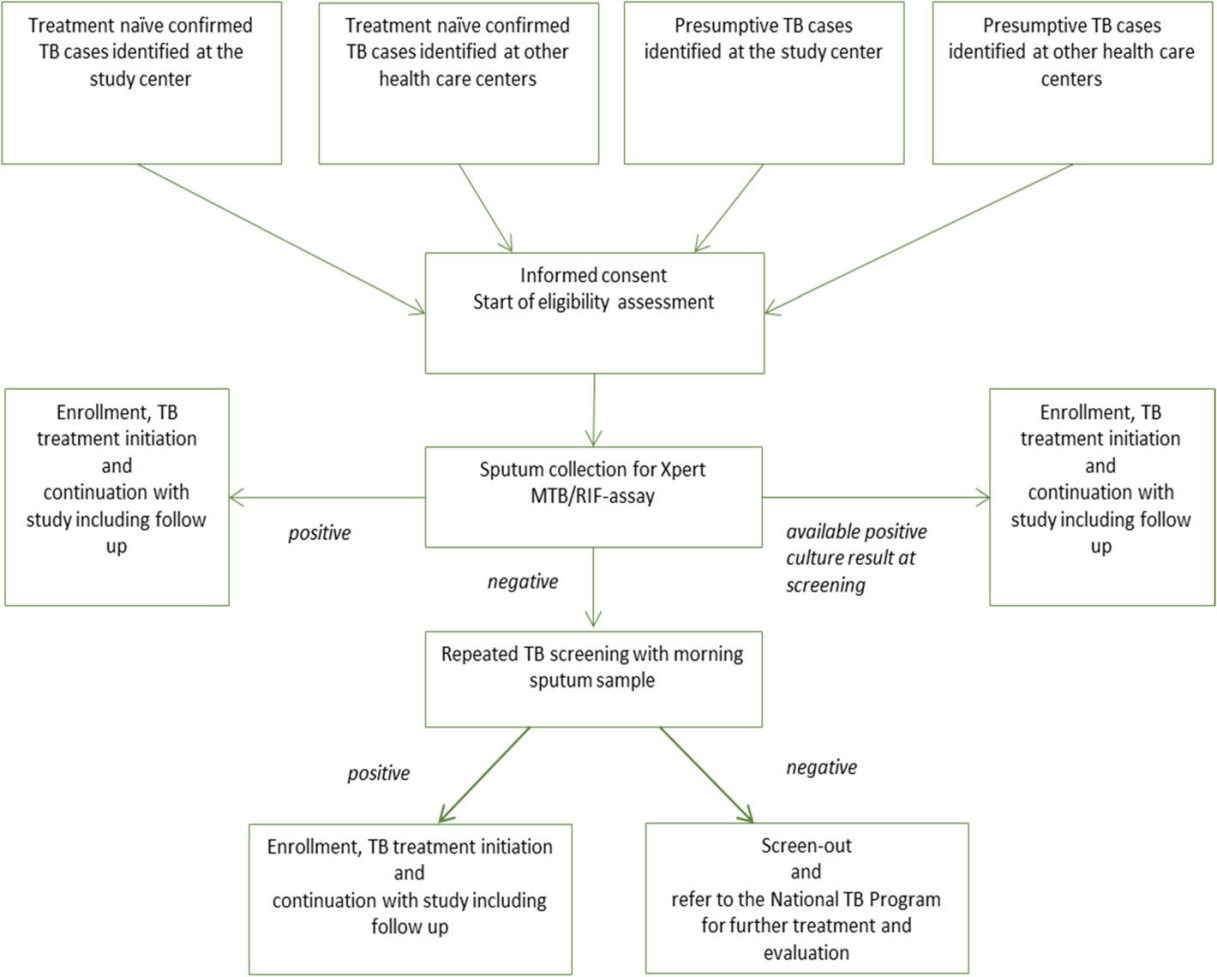
Screening algorithm

We will conduct a prospective, multi-country, multi-centre, observational cohort study and recruit up to 1.600 pulmonary TB patients at the time of TB diagnosis who will be followed during and after TB treatment. At enrolment, TB diagnosis is based on sputum tested positive for the presence of MTB either by Xpert MTB/RIF (or Ultra) or culture methods (Fig. 1). At baseline and at defined study visits (see Schedule of Events (SOE) Table 1) clinical data, data on risk factors, information on comorbidities, socio-economic data, as well as biological samples (sputum, urine and blood) will be collected from all participants. This large cohort will therefore serve as a platform for all nested research activities related to immunological, microbiological and socio-economic studies as well as future therapeutic interventions (Additional file 2).

**Table 1:** Schedule of Events (SOE)

Activities	Screening	Baseline	Day 14	Month 2	Month 4	Month 6	Month 9	Month 12	Month 18	Month 24
Visits										
Eligibility assessment	x									
Xpert MTB/RIF (Ultra) assay	x									
Sputum smear and culture	x	x		x	x	x	(x)	(x)	(x)	(x)
Drug sensitivity testing		x								
Demographic data		x								
Clinical examination		x	x	x	x	x	x	x	x	x
Chest X-ray		x				x				x
Lung function assessment		x		x		x	x	x	x	x
ECG		x						x		x
Medical history (update)		x	x	x	x	x	x	x	x	x
TB treatment and adherence			x	x	x	x	(x)	(x)	(x)	(x)
Quality of life/patient’s cost questionnaire		x		x		x		x		x
HIV test (CD4 count)		x						x		x
Hepatitis B serology		x								
Haematology and Biochemistry		x				x		x		x
Urine dip stick test		x		x	x	x				
Blood storage for nested research studies		x	x	x	x	x	x	x		
Sputum storage for nested research studies		x	x	x	x	x	(x)	(x)	(x)	(x)
Urine storage for nested research studies		x		x	x	x	(x)	(x)	(x)	(x)

### Participant eligibility

For enrolment, each study participant must meet all of the inclusion criteria and none of the exclusion criteria summarized in Table 2.

**Table 2:** Inclusion and exclusion criteria

	Inclusion criteria	Exclusion criteria
1	At least one sputum sample tested positive for MTB by Xpert MTB/RIF assay in the study clinic/study laboratory or at least one sputum sample tested positive by culture methods in study laboratory or other TB laboratory	Anti-TB treatment in the last 6 months^a^
2	Be ≥18 years of age	Incapacity to produce and provide two sputum samples of sufficient volume and quality^b^
3	Willing to provide a written consent or witnessed oral consent in the case of illiteracy for participation in the study, prior to patient’s first sample or other study-specific data being collected	Severe medical or psychiatric condition which in the opinion of the site investigator or designee, might interfere with the ability to give true informed consent and to adhere to the study requirements
4	Willing to be tested for HIV infection	Currently imprisoned
5	Agreeing to the collection and storage of blood, urine, and sputum specimens	Taking part in investigational product trials related to TB and/or lung diseases
6	Willing to start anti-TB treatment after TB diagnosis	
7	Living within the study area and willing to inform the study team of any change of address during the treatment and follow up period	

^a^To ensure that no treatment failure are recruited into (immunological or genetic) marker studies

^b^To ensure sufficient amount of sputum for host- and mycobacterial marker studies

### Duration of study

Recruitment commenced in September 2017 and is expected to continue until September 2019. Follow-up will last for a minimum of 24 months in all participants to ensure that any loss in reported lung function will have become chronic during follow-up [28]. Further, the chronicity and final degree of long term costs and comorbidities resulting from TB disease or treatment shall be confirmed by a follow up period of at least 24 months length. End of study is expected in September 2021.

### Primary outcome and study endpoints

The primary outcome is a proportion of TB patients with severe pulmonary function impairment (restrictive, obstructive or mixed ventilation impairment), measured by spirometry at 24 months from TB treatment initiation and compared to national reference values. Long term lung function impairment is considered a clinically meaningful condition contributing to long term morbidity and mortality in former TB patients.

Amongst secondary outcomes are: the proportion of subjects with any impairment of pulmonary function as well as severity and type of impairment at TB diagnosis and 2, 6, 12 and 18 months; proportion of subjects with liquid and solid MTB culture conversion at different time points during or at the end of anti-TB treatment; the frequency of all-cause hospitalization episodes and all-cause mortality during follow-up of 24 months; the proportion of subjects with recurrent TB and; the proportion of subjects suffering from ongoing non-pulmonary comorbidities which are related to TB disease and treatment at different time points during 24 months of follow-up.

Studies regarding host immunology- and pathogen-associated risk factors and markers are planned as explorative, nested, case-control studies. Sample selection will be based on specific outcomes and risk factors present in the study participants.

Main outcomes studied in the context of socio-economic research are the proportion of subjects incurring catastrophic cost due to TB by the end of treatment; changes in income within follow up period; and scales for quality of life and general disability at treatment start, end of treatment and at follow-up. The cost of providing TB care and treatment, will be estimated from the health provider perspective, for both drug sensitive and drug resistant TB.

### Sample size

We hypothesized that the prevalence of the primary endpoint - severe lung impairment at 24 months period of follow up after TB diagnosis (conservative estimation based on a literature review) will be 15%. A sample size of 1.600 patients allows the estimation of the primary end point in the total cohort with a precision (half width of the 95% confidence interval) of about 1.75%. In addition, the prevalence within specific subgroups can be estimated adequately, e.g. with a precision between 2.5 and 7.8% in subgroups with a proportion of 50% (e.g. HIV) and 5% (e.g. diabetes) of the total cohort, respectively. Moreover, based on a pre-set alpha of 5% (two-sided) the sample size allows the detection of risk factors causing a duplication of the prevalence of severe lung impairment with a power of at least 80%, even for risk factors with a low prevalence of 5% in the total cohort. Depending on the availability and quality of patient’s end-point data, or if the prevalence of the primary outcome is less than 15%, it may be necessary to recruit more or less participants into the study.

### Recruitment and screening

Recruitment takes place at the research sites and health care facilities through collaboration with National TB Program (NTP), and in the communities which might be enhanced by individual and community awareness through advertisement, posters, radio announcements, and community sensitization campaigns. Each study site implements its own strategy to enhance recruitment.

### Study procedures

Clinical and pulmonary assessments, biological sample collections and collection of socio-economic data will be performed according to the pre-defined SOE. All enrolled patients will be treated within the NTP and receive anti-TB treatment according to the local national guidelines.

#### Clinical assessments and procedures

We will assess the pulmonary outcome, co-morbidities and clinical risk factors using a number of methods. These include: spirometry using the EasyOne or EasyOnPC (ndd Medical Technologies, Inc., Zurich, Switzerland), Electrocardiogram (ECG), 6 min walking test (6MWT), chest X-ray, St. George’s respiratory questionnaire, blood analysis (haematology, biochemistry including HbA1c, HIV/CD4, hepatitis B), urinalysis and risk-factors questionnaires (risk behaviour, environmental and occupational risk factors, among others). In addition, household air pollution is estimated by measuring participant’s breath carbon monoxide (CO) output (exhaled CO levels), which is collected using a handheld breath CO monitor - The Micro+™ Smokerlyzer® (Bedfont® Scientific Ltd., UK).

#### Laboratory procedures and testing

All laboratory procedures (apart from some specific assays planned for investigation of host-immune response or pathogen characteristics not locally available) will be performed at each study site and at the designated site laboratories under the supervision of the study principal investigators and using unified standard operating procedures. In the study TB labs we will carry out standard TB diagnostic procedures including smear, Xpert MTB/RIF or Ultra assay (Cepheid Inc., USA), liquid and solid culture, drug resistance testing in culture and molecular speciation testing. For analysis of pathogen-related risk factors, we will perform a number of pilot studies including characterization of MTB strains by next generation whole genome sequencing, identification of mixed infections and minority subpopulations as well as capture the bacterial load, e.g. using molecular methods to monitor treatment response. A number of different assays will be performed to study in detail the host response to TB treatment and individual predisposing risk factors for long term lung outcome on stored samples, such as (but not limited to) sputum and whole blood transcriptomics, flow cytometry, cytokine assays and host-genetic analysis.

#### Assessments related to socio-economic consequences and quality of life

All patients will be interviewed by trained study staff using a novel version of the WHO’s generic TB patient cost survey instrument [27] which has been adapted for both the longitudinal study design and local context of each setting. The instrument includes questions about direct out-of-pocket costs (net of reimbursement) of medical care, transport, food and accommodation during health seeking, indirect costs such as income loss and socio-economic coping mechanisms (e.g. taking a loan, selling assets or property or taking children out of school). Moreover, the instrument collects information about health insurance coverage and reimbursements and social welfare or paid sick leave received. A standardized short-form 36 item health survey (SF-36) combined with a PIQ-6™ (4 items) will simultaneously measure overall health, pain severity and the impact on functional health and well-being. In addition to the above we will also be using the Sheehan Disability scale (5 items) to assess health status impairment associated with TB and Kessler Psychological Distress scale (K10), a 10 item questionnaire, to measure distress based on questions about anxiety and depressive symptoms. These are all standardized validated questionnaires that have been widely used in TB studies and are simple to administer and repeatable over time. The patient cost and quality of life questionnaires will be completed at baseline and at pre-defined visits (e.g. 2, 6, 12 and 24 months). Health system costs will be based on actual patient usage with unit costs assigned, but will be collected later for the 24 month period. Resource utilization and treatment outcomes will be collected using clinical records, health facilities records, price lists and reports.

### Sample collection and storage

Blood, sputum and urine specimens are obtained from each patient at different time points for analysis and storage. The SOE is aligned with The RePORT International cohort and other TB cohorts to enable future research collaborations with other TB research consortia and facilitate comparability of outcome data across different cohorts. The researchers who manage or have access to human specimen data are legally and ethically obliged to protect these data according to international standards. Specific samples, e.g. TB strains, will be shipped to collaborating institutions and stored in their laboratories for further analysis.

Stored specimens will also be accessible for evaluation of future emerging TB diagnostics, genetic markers and biomarkers, provided informed consent and ethical approval for future use are available. For any additional investigations not explicitly mentioned in the original study protocol, a request for study amendment will be submitted to the relevant ethics committees.

### Data management and statistical analysis

A study specific database has been developed using a web-based Clinical Data Management System - Open Clinica®, for data entry and data cleaning, supervised by the lead data manager at LMU. All data collected at each performed visit must be entered in the respective electronic case report forms (eCRFs) provided in the data base.

The statistical analysis of this observational cohort study will be exploratory and use descriptive as well as inferential statistical methods. The statistical analyses will primarily be based on all enrolled patients with valid data available. Preliminary data sets will be analysed along the study in the frame of interim analyses, with the final analysis being completed once the database is locked.

### Study monitoring

Study activities related to data generation, recording and management processes will be monitored on a regular basis. Two external monitors from coordinating institutions have been assigned to this task. These clinical monitors will follow the study closely, visit the sites at regular intervals and will be in contact by phone and written communication, as required. In addition to close supervision by the external monitors as well as by a local supervisor, one internal clinical monitor is supervising the procedures in the study clinics at each study site in the context of a continuing clinical monitoring training which includes workshops and distance learning modules. Additionally, an External Advisory Board consisting of six representatives of NTPs, local and international policy makers as well as relevant (clinical) scientists in the field of TB research was created to regularly evaluate the study activities and research progress as well as support the process in scientific decision making with regards to the global TB research agenda.

### Ethical considerations

This study is performed in accordance with the study protocol, the Declaration of Helsinki (October 2013) and the WHO Handbook for Good Clinical Research Practice (July 2002) as well as any other applicable national and other regulatory guidelines. The protocol, Informed Consent Form (ICF), eCRFs and other relevant study documents were reviewed and approved by all respective Ethical Committees at each study site and also at coordinating institutions. All participants sign an ICF prior to enrolment in the study. For each enrolled subject, we will assign a unique study identification number, which will be used to identify the subject’s data and sample within the project. The eCRFs and other study documents do not include any identifying information linked to the study ID in order to maintain confidentiality for all records and data of the participants.

## Discussion

Current guidelines do not foresee specific monitoring or treatment of pulmonary injury related to TB, nor has disability due to long term loss of lung function or other sequelae been included in current global estimates of the total burden of disease. Social support for TB patients rarely consider the period after cure has been declared. As NTP in most countries do not follow up patients after “cure”, the extent of the long-term complications and their impact on individuals and households so far remain unknown.

The present lack of knowledge regarding the pathogenic mechanisms or clinical co-factors causing adverse outcomes in TB hinders the development and evaluation of interventions to reduce the overall TB disease burden or costs related to TB. Additionally, the lack of current, geographically relevant information about the economic impact of TB and the likely costs to national health systems pose an obstacle to efficient resource allocation both within TB programs and across competing health and social care priorities. Studies, such as TB Sequel, examining long term disability and associated risk factors, income loss, and treatment-related costs are crucial to inform the policy changes needed to address poor long-term TB outcome and catastrophic costs burden. Compared to other TB cohort studies TB Sequel will follow patients for at least 24 months which enables us to report lung outcome when any impairment related to TB disease has stabilized, thus when it can be assumed to have become chronic. Likewise the characteristics of ongoing socio-economic consequences and disease burden can be studied in those patients with relevant chronic lung or other TB related injury for at least 24 months. This allows for new estimates of TB disease burden which go beyond microbiological cure. Due to sample size and selection of different study sites across Africa we will be able to describe the influence of important potential risk factors on lung outcome such as infection with an MDR-TB strain, HIV-coinfection and comorbidities like COPD and diabetes but also the relevance of different genetic and socio-economic background. On the other hand, the follow-up of only 24 months precludes the investigation of interrelation of pulmonary TB with pre-existing and especially newly developed comorbidities as well as the influence of TB on long term survival and socio-economic status. Another limitation of the study is that the investigators have no influence on the composition of and adherence to anti-TB (and anti-retroviral) treatment and individual treatment regimens might have a different influence on lung outcome. On each study visit, co-medication and drug adherence are documented in order to enable sub-group analyses as well as to control for it during analysis of other risk factors for poor treatment outcomes. Finally, the capacities for adequate treatment and follow up for the majority of diagnosed comorbidities are currently limited or even not available at most study settings.

Thus, the TB Sequel Project is an integral part of an overall strategy to fill a knowledge gap needed to improve TB treatment and outcomes globally. It is an interdisciplinary project that combines multiple components: research, capacity building and translation of evidence into policies. All research sites will receive training and are included in capacity building activities (both in human resources and infrastructure) which creates an enabling environment for research and increases competencies in diagnosis and management of TB and lung health with direct benefit for future TB patients.

### Dissemination of results

We anticipate that the results of this study will be relevant to a broader research community, health care providers and policy makers. Hence, we will disseminate the findings of this study through different channels, such as TB Sequel web page (www.tbsequel.org), scientific articles in international peer-reviewed journals, presentations at national and international conferences, social media and policy briefs. To further increase the impact of our findings, national and supra-national stakeholders and decision makers, including the World Health Organization, were consulted early in the study planning process and results will be regularly shared with these institutions to inform future treatment guidelines and national health policies. The dissemination of research results will be an integral part of the TB Sequel project which also focuses on collaboration strengthening through: developing new and intensifying existing links between involved research institutions by integrating and harmonizing common activities to create an enabling environment at African research institutions for the exchange of knowledge; participation of local, regional and international stakeholders in the project to facilitate translation of evidence from the TB Sequel study into policy and practice’ and finally, the collaboration with other research networks such as PanACEA, The RePORT International, German Center for Infection Research (DZIF) projects and RESULTS Africa to ensure the relevance and impact of TB Sequel research activities**.**

## Additional files

Additional file 1: TB Sequel Project partners and research sites. (DOCX 276 kb)

Additional file 2: Overview diagram depicting the interrelation of the main TB-cohort and embedded sub-studies. (DOCX 91 kb)

## Abbreviations

AIDS: Acquired immune deficiency syndrome
CO: Carbon monoxide
COPD: Chronic obstructive pulmonary disease
DZIF: German Center for Infection Research
ECG: Electrocardiography
eCRF: Electronic clinical report form
FEV1: Forced expiratory volume in the first second
HIV: Human immunodeficiency virus
ICF: Informed consent form
IRIS: Immune reconstitution inflammatory syndrome
MTB: Mycobacterium tuberculosis
NTP: National TB Program
SOE: Schedule of events
TAM TB: T-cell activation marker-tuberculosis
TB: Tuberculosis
WHO: World Health Organization
