# Supplementary figures and images for: TB sequel: incidence, pathogenesis and risk factors of long-term medical and social sequelae of pulmonary TB – a study protocol
Source: BMC Pulm Med. 2019 Jan 7;19:4. doi: 10.1186/s12890-018-0777-3 (PMC6323671; doi:10.1186/s12890-018-0777-3)

**Additional file 1**

TB Sequel Project partners and research sites

**
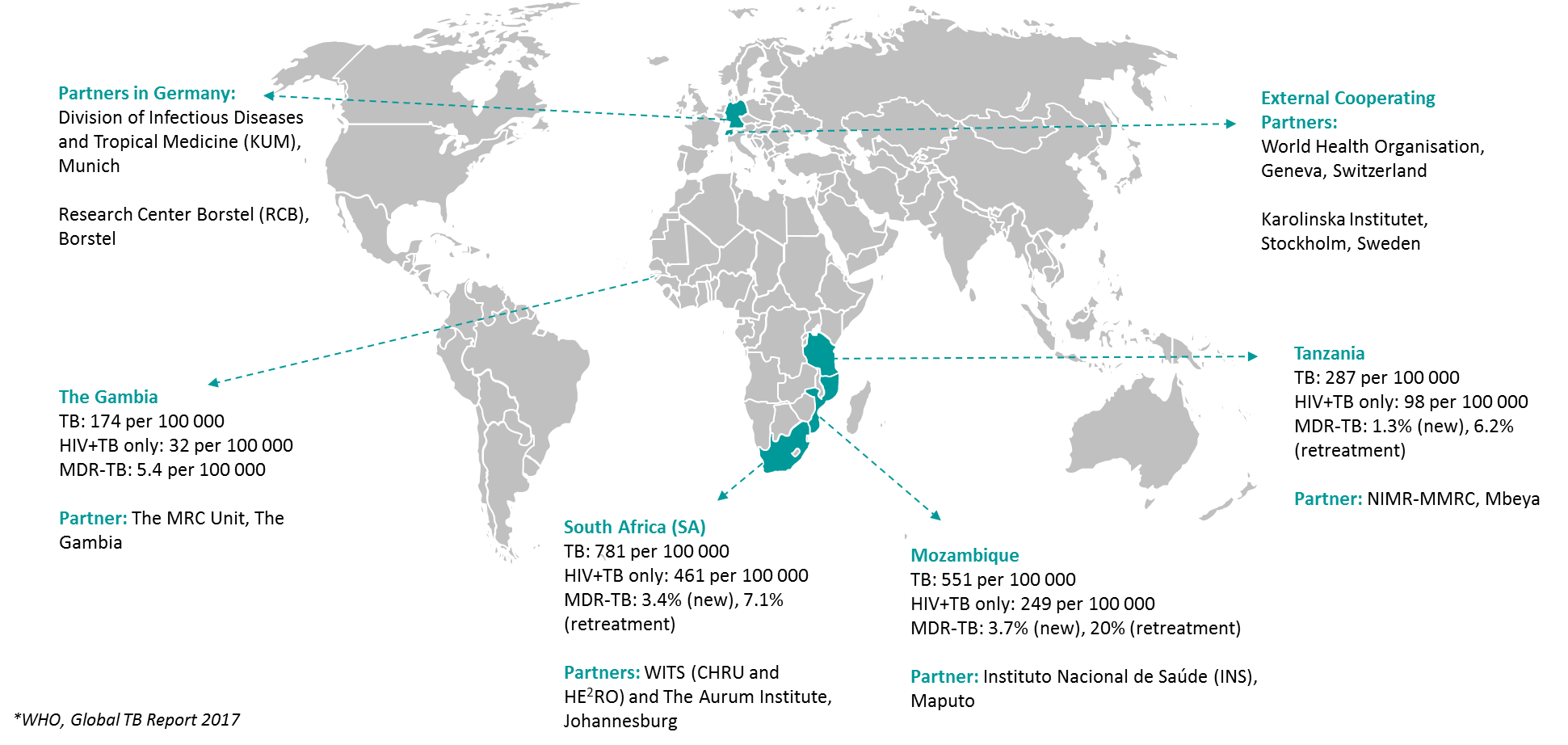
**

Supplement: Supplementary file 1 — TB Sequel Project partners and research sites. (DOCX 276 kb) [file 12890_2018_777_MOESM1_ESM.docx]
