# Supplementary material for: TB sequel: incidence, pathogenesis and risk factors of long-term medical and social sequelae of pulmonary TB – a study protocol
Source: BMC Pulm Med. 2019 Jan 7;19:4. doi: 10.1186/s12890-018-0777-3 (PMC6323671; doi:10.1186/s12890-018-0777-3)
Supplement: Supplementary file 2 — Overview diagram depicting the interrelation of the main TB-cohort and embedded sub-studies. (DOCX 91 kb) [file 12890_2018_777_MOESM2_ESM.docx]

**Additional file 2**

Overview diagram depicting the interrelation of the main TB-cohort and embedded sub-studies

**
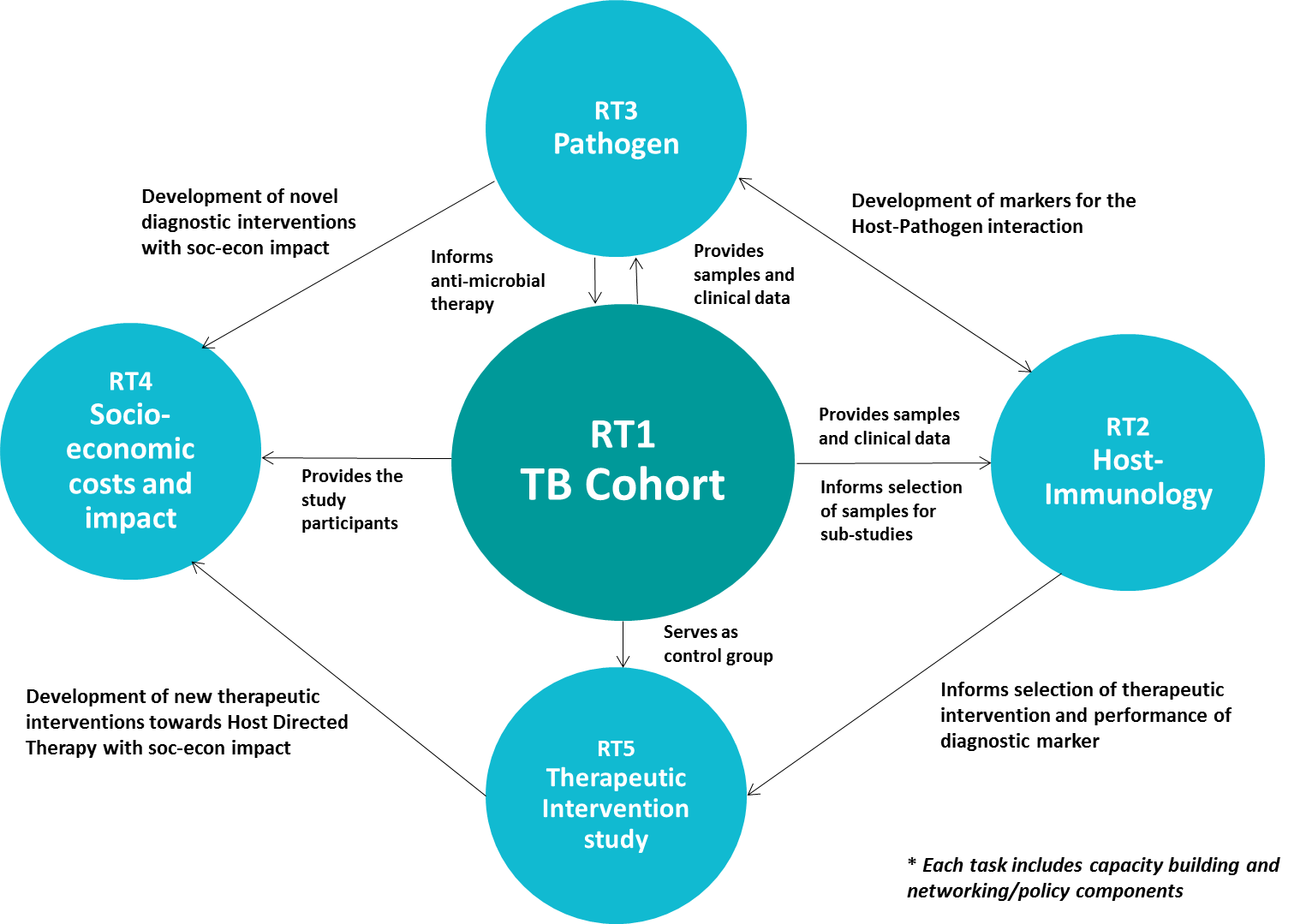
**
